# Long non-coding rnas as key modulators of the immune microenvironment in hepatocellular carcinoma: implications for Immunotherapy

**DOI:** 10.3389/fimmu.2025.1523190

**Published:** 2025-04-25

**Authors:** Siqi Zhao, Fei Chen, Lingyu Hu, Xiaoping Li, Zhaofeng Gao, Minjie Chen, Xiaoguang Wang, Zhengwei Song

**Affiliations:** Department of Surgery, the Second Affiliated Hospital of Jiaxing University, Jiaxing, Zhejiang, China

**Keywords:** lncRNAs, HCC, immune microenvironment, immunotherapy, immune evasion

## Abstract

Hepatocellular carcinoma (HCC) represents a major global health challenge, characterized by its complex immune microenvironment that plays a pivotal role in tumor progression and therapeutic response. Long non-coding RNAs (lncRNAs) have emerged as critical regulators of various biological processes, including gene expression and immune cell function. This review explores the multifaceted roles of lncRNAs in modulating the immune microenvironment of HCC. We discuss how lncRNAs influence the infiltration and activation of immune cells, shape cytokine profiles, and regulate immune checkpoint molecules, thereby affecting the tumor’s immunogenicity and response to immunotherapy. Furthermore, we highlight specific lncRNAs implicated in immune evasion mechanisms and their potential as biomarkers and therapeutic targets. By elucidating the intricate interplay between lncRNAs and the immune landscape in HCC, this review aims to provide insights into novel strategies for enhancing immunotherapeutic efficacy and improving patient outcomes.

## 1 Introduction

HCC is the most common type of primary liver cancer and ranks as one of the leading causes of cancer-related mortality worldwide (1, 2). Its development is often associated with underlying liver diseases, including chronic hepatitis B and C infections, cirrhosis, and metabolic disorders (3–5). Despite advances in surgical and systemic therapies, the prognosis for patients with HCC remains poor, largely due to the tumor’s aggressive nature and its ability to evade the host immune response (6).

The tumor microenvironment (TME) plays a crucial role in HCC progression and therapeutic resistance (7). It is composed of various cell types, including tumor cells, immune cells, fibroblasts, and endothelial cells, all of which interact dynamically to influence tumor behavior (7–9). These immune cells can either promote anti-tumor immunity or facilitate tumor growth through immunosuppressive mechanisms, contributing to the challenges faced in effective treatment (8, 10).

At present, cancer immunotherapies such as immune checkpoint inhibitors (ICIs) (such as anti-PD-1/PD-L1 monoclonal antibody) and CAR-T cell therapy have made breakthroughs in a variety of tumors, but their efficacy in HCC is still limited to a certain extent (11–13). Although checkpoint inhibitors partially improve the survival of patients with advanced HCC, their overall response rate is less than 20% and they are prone to secondary drug resistance (14). CAR-T therapy is effective in hematologic tumors, but progress is slow due to the high heterogeneity of HCC, immunosuppressive microenvironment and physical barrier of solid tumors (15). In addition, HCC is often accompanied by chronic inflammation and cirrhosis, leading to depletion of immune cell function and increased infiltration of immunosuppressive cells (such as Treg and MDSC), further weakening treatment response (8). These limitations highlight the urgent need for in-depth analysis of HCC-specific immune regulatory mechanisms, such as lncrNA-mediated immune escape, to develop combination strategies.

Recent studies have identified lncRNAs as critical regulators of gene expression and cellular processes in cancer (16, 17). These RNA molecules, which are over 200 nucleotides in length and do not code for proteins, have been shown to play diverse roles in modulating cellular behavior, including apoptosis, proliferation, and immune responses (18, 19). Emerging evidence suggests that lncRNAs are involved in shaping the immune microenvironment of various tumors, including HCC, by regulating the functions of immune cells and influencing the expression of immune-related genes (20).

In this review, we will explore the intricate relationships between lncRNAs and the immune microenvironment in HCC. We will discuss how lncRNAs contribute to immune cell infiltration, modulation of cytokine secretion, and regulation of immune checkpoints, thereby impacting the tumor’s immunogenicity and response to therapies. By understanding these mechanisms, we aim to highlight the potential of lncRNAs as biomarkers and therapeutic targets in HCC, paving the way for novel strategies to enhance immunotherapy outcomes.

## 2 HCC and immune microenvironment

The immune microenvironment in HCC is a complex network that significantly influences tumor progression and therapeutic outcomes (3, 21). Comprised of various immune and stromal cell types, the immune landscape of HCC is characterized by both pro-tumor and anti-tumor activities. A critical feature of this microenvironment is its ability to foster immune evasion, which allows tumors to grow and metastasize (7, 22).

In HCC, diverse immune cell populations, including T cells, natural killer (NK) cells, dendritic cells, and myeloid-derived suppressor cells (MDSCs), contribute to the tumor immune landscape. The balance between these cell types can determine the effectiveness of the immune response against tumors. A high infiltration of MDSCs and regulatory T cells (Tregs) is often associated with poor prognosis, as these cells can suppress the activity of effector T cells and promote an immunosuppressive microenvironment (23, 24). Conversely, a robust presence of activated cytotoxic T lymphocytes (CTLs) and NK cells is linked to better patient outcomes, as these cells can effectively target and destroy tumor cells (25–27).

The immune microenvironment in HCC is heavily influenced by the local cytokine milieu, which can dictate the behavior of immune and tumor cells (28, 29). Pro-inflammatory cytokines such as IL-6 and TNF-α often dominate the microenvironment, promoting tumor proliferation and facilitating immune evasion (30, 31). These cytokines can enhance the recruitment of immunosuppressive cells while inhibiting the function of effector immune cells. Moreover, a dysregulated cytokine profile can lead to chronic inflammation, which is a hallmark of HCC development. This chronic inflammatory state not only supports tumor growth but also contributes to the creation of an immune environment that is unfavorable for effective anti-tumor responses (32, 33).

Immune checkpoint molecules, including PD1, PD-L1, and CTLA-4, play a crucial role in regulating immune responses in HCC (34, 35). The expression of these checkpoints can be upregulated in response to theTME, leading to diminished T cell activation and function (36).

The upregulation of PD-L1 on tumor cells, in particular, has been associated with poor clinical outcomes in HCC, as it enables cancer cells to evade immune detection (37, 38). ICIs targeting PD1 and CTLA-4 have shown promise in clinical settings, offering new avenues for immunotherapy in HCC (39, 40). However, the effectiveness of these therapies can be hindered by the complexity of the immune microenvironment and the various mechanisms of immune evasion employed by HCC.

**Figure 1:**
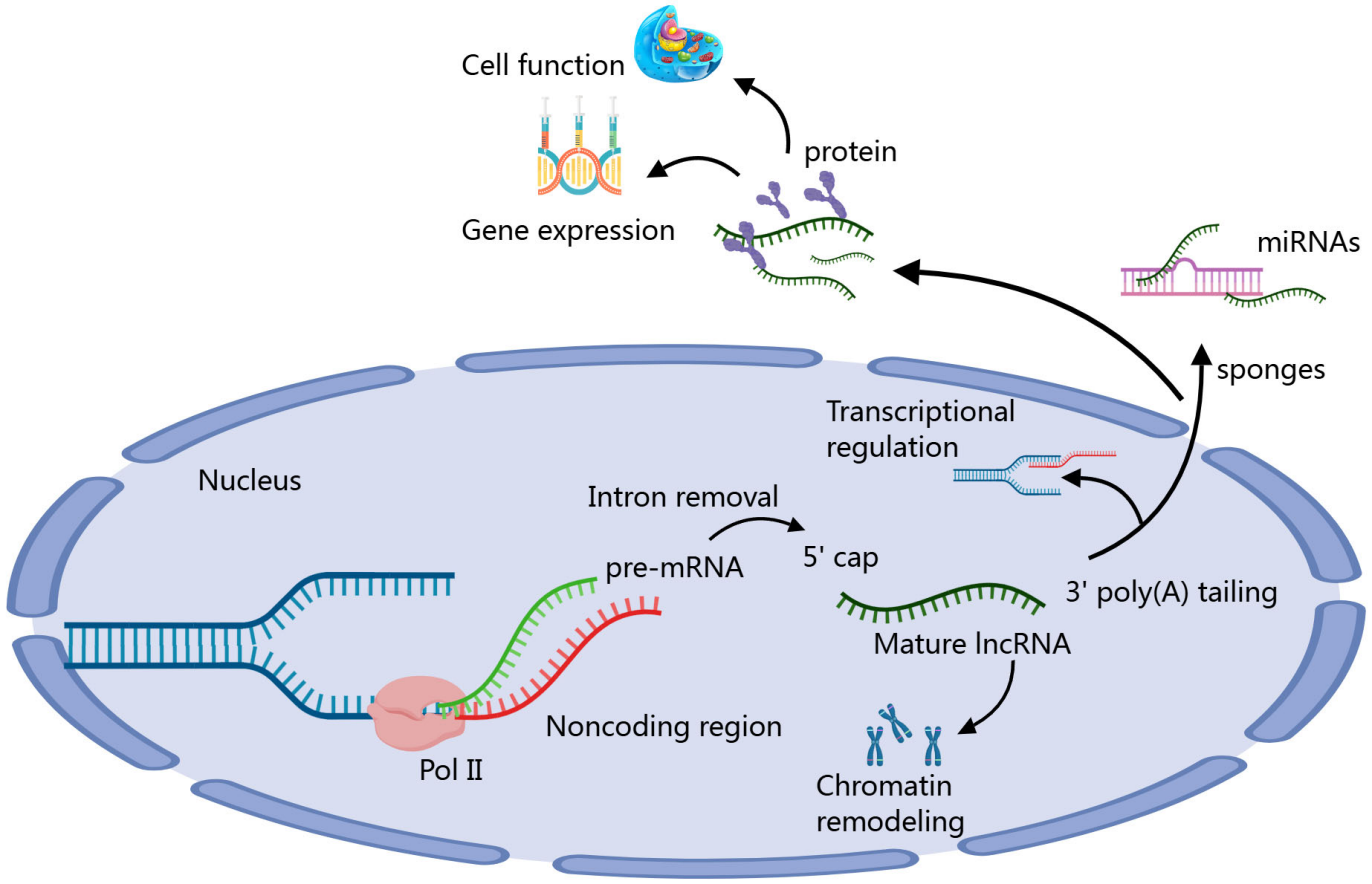
The formation process and function diagram of lncRNAs (37, 38).

lncRNAs have emerged as important regulators of the immune microenvironment in HCC, influencing various aspects of immune cell behavior and interactions (41). The formation process of lncRNAs involves several steps. First, lncRNAs are transcribed from genomic DNA by RNA polymerase II, typically existing adjacent to or independently from protein-coding genes. After transcription, lncRNAs undergo processing, including 5’ capping, 3’ polyadenylation, and splicing, to become mature. Mature lncRNAs can function in the nucleus or cytoplasm, regulating gene expression and cellular functions through interactions with DNA, RNA, or proteins. They can act as “sponges” to absorb specific miRNAs or participate in transcriptional regulation and chromatin remodeling, thus playing various important biological roles (**Figure 1**) (42, 43). These lncRNAs can modulate the infiltration and activity of immune cells, alter cytokine production, and regulate immune checkpoint expression (44). For instance, certain lncRNAs have been shown to enhance the recruitment of immunosuppressive cells, such as MDSCs and Tregs, thereby promoting an immunosuppressive environment conducive to tumor growth. Conversely, other lncRNAs may activate effector immune responses by enhancing the function of cytotoxic T cells and NK cells (45–47). By shaping the immune landscape, lncRNAs play a critical role in determining the efficacy of immunotherapies in HCC. Targeting specific lncRNAs could offer a novel approach to reprogram the immune microenvironment, restore anti-tumor immunity, and improve patient responses to existing therapies (48). Thus, further investigation into the functional roles of lncRNAs in HCC’s immune landscape is essential for developing innovative therapeutic strategies.

## 3 LncRNA regulation of the immune Microenvironment in HCC

### 3.1 Regulation of immune cells by lncRNAs in HCC

**Figure 2:**
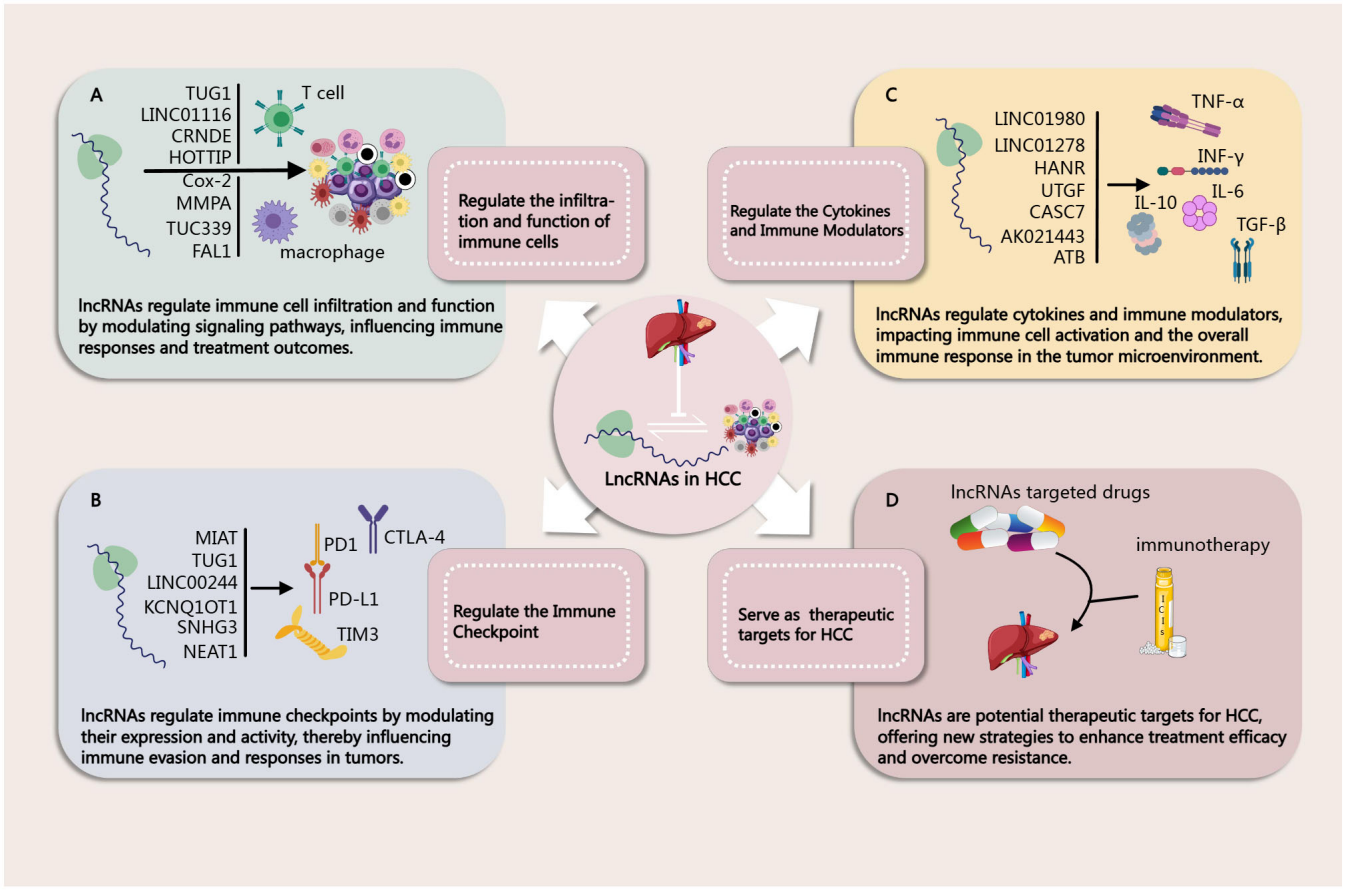
**(A)** Regulation of immune Cells by lncRNAs in HCC (46, 49, 50, 52, 65–67, 72). **(B)** Regulation of Immune Checkpoint by lncRNAs in HCC (44, 46, 47, 63, 91, 92). **(C)** Regulation of Cytokines and Immune Modulators by lncRNAs in HCC (94–96, 98–100, 106). **(D)** lncRNAs as Biomarkers in HCC Immunotherapy (46, 47, 65, 96).

As mentioned above, in the complex immune microenvironment of HCC, lncRNAs play a crucial regulatory role. Recent studies have demonstrated that lncRNAs not only participate in the biological processes of tumor cells but also significantly influence the immune evasion mechanisms of HCC by modulating the functions and interactions of immune cells (**Figure 2A**). These lncRNAs can shape the TME through various mechanisms, including affecting the differentiation, activation, and functional status of immune cells, as well as regulating intercellular signaling. In this chapter, we will delve into the regulatory mechanisms of lncRNAs on specific immune cells, such as T cells, B cells, macrophages, and dendritic cells, analyze their potential roles in HCC, and discuss the prospects of targeting them for therapeutic interventions.

#### 3.1.1 T cell

T cells play a crucial role in the immune response of HCC, and lncRNAs regulate T cell function and activity through various mechanisms. They influence T cell development, differentiation, and effector functions, thereby altering the immune balance within the TME. Previous studies have demonstrated the overexpression of several oncogenic lncRNAs in HCC, including TUG1, LINC01116, CRNDE, MIAT, E2F1, LINC01132, and Lnc-Tim3. These lncRNAs can influence T cell activity and function through various pathways or cascading effects, thereby promoting tumor progression. lncRNAs often modulate T cell co-stimulation by regulating downstream miRNAs. Research has shown that NEAT1 and Tim-3 are significantly upregulated in the PBMCs of HCC patients. Downregulation of NEAT1 can inhibit the apoptosis of CD8+ T cells and enhance their cytolytic activity against HCC cells, while interference with miR-155 shows the opposite effect, promoting the upregulation of Tim-3. This study confirms the binding and interaction of NEAT1 with miR-155 in CD8+ T cells. By regulating the miR-155/Tim-3 pathway, downregulation of NEAT1 can inhibit CD8+ T cell apoptosis and enhance their cytolytic activity against HCC, indicating that NEAT1 is an effective target for improving immunotherapy outcomes (49). Lnc-Tim3 can specifically bind to Tim-3, preventing its interaction with Bat3, thereby inhibiting downstream signaling in the Lck/NFAT1/AP-1 pathway, leading to the nuclear localization of Bat3. This enhances the transcriptional activation of pro-apoptotic genes (such as MDM2 and Bcl-2) by p300-dependent p53 and RelA, promoting T cell exhaustion. This suggests that Lnc-Tim3 and its associated signaling pathways may impact the effectiveness of cancer therapies targeting the adaptive immune system (50).

Some lncRNAs can affect T cell function by regulating the expression of immune checkpoints. For instance, TUG1 is upregulated in HCC due to METTL3-mediated m6A modification. Knockdown of TUG1 can inhibit tumor growth and metastasis while increasing the infiltration of CD8+ T cells and M1 macrophages in tumors. TUG1 activates CD8+ T cells through PD-L1 and enhances macrophage phagocytosis via CD47. Mechanistically, TUG1 acts as a “sponge” for miR-141 and miR-340 to regulate the expression of PD-L1 and CD47 while interacting with YBX1 to promote their transcription. Clinical data also indicate a positive correlation between TUG1 and PD-L1 and CD47 in HCC tissues. Co-treatment with Tug1-siRNA and Pdl1 antibodies effectively inhibits tumor growth (51). Additionally, MIAT promotes STAT3 expression by negatively regulating miR-411-5p, thereby increasing the transcription level of PD-L1. Knockdown of MIAT enhances T cell cytotoxicity against HCC cells, and inhibiting miR-411-5p can reverse the decrease in STAT3 and PD-L1 expression caused by MIAT knockdown (52). The lncRNA CTD-2510F5.4 is significantly upregulated under hypoxic conditions and is expressed in Huh7 and HepG2 cells. Overexpression of CTD-2510F5.4 promotes the proliferation, invasion, and metastasis of HCC cells. Patients with high expression of CTD-2510F5.4 have poor prognoses and higher TP53 mutation rates, increased infiltration of immunosuppressive Tregs, and elevated expression of immune checkpoint molecules, leading to impaired immune function. Notably, patients with low expression of CTD-2510F5.4 show higher sensitivity to immunotherapy and anti-angiogenic therapy, while those with high expression respond better to chemotherapy (53).

Some lncRNAs can also influence the immune microenvironment by regulating tumor metabolism. For example, LINC01116 inhibits RAD18-mediated ubiquitination, stabilizing the EWSR1 protein and upregulating the expression of PPARA and long-chain fatty acid transporter FABP1, leading cancer cells to preferentially uptake linoleic acid and other long-chain fatty acids, resulting in T cell dysfunction and malignant progression of HCC. In mouse models, blocking LINC01116 enhances the efficacy of anti-PD1 therapy, increasing the infiltration of cytotoxic T cells and reducing the proportion of exhausted T cells (54).

Additionally, some lncRNAs can influence T cell infiltration and function through various cascading pathways, thereby affecting tumor progression. CRNDE is primarily found in malignant tumor cells and promotes tumor cell growth, recruits a large number of granulocyte-MDSCs (G-MDSCs), and restricts T cell infiltration, thereby facilitating HCC progression. Mechanistically, CRNDE binds to TLR3, activating the NF-κB signaling pathway and increasing the secretion of CXCL3. Knockdown of CRNDE significantly reduces G-MDSC accumulation and enhances T cell infiltration in the TME (55). LINC01132 has been identified as a novel oncogenic lncRNA, with its expression significantly higher in HCC tumor tissues compared to normal tissues, correlating with poor survival rates in patients, primarily driven by copy number amplification. Functional studies indicate that overexpression of LINC01132 promotes cell growth, proliferation, invasion, and metastasis. Mechanistically, LINC01132 enhances DPP4 expression through physical interaction with NRF1, acting as an oncogenic driver. Furthermore, silencing LINC01132 can induce CD8+ T cell infiltration, and when combined with anti-PD-L1 treatment, can improve anti-tumor immunity, suggesting that LINC01132 may serve as a novel immunotherapeutic target in HCC. Therefore, LINC01132 promotes HCC development through the NRF1/DPP4 axis, and targeting LINC01132 may enhance the efficacy of anti-PD-L1 immunotherapy (56).

These findings highlight the complex roles of lncRNAs in the immune microenvironment, suggesting that developing therapeutic strategies targeting specific lncRNAs may provide new avenues to enhance the efficacy of immunotherapy in HCC. Further related studies are summarized in **Table 1**.

**Table 1:** Regulation of T Cells by lncRNAs in HCC.

LncRNAs	Mechanism and Effect	Clinical significance	Ref
TUG1	TUG1, upregulated by METTL3-mediated m6A, regulates PD-L1 and CD47 by sponging miR-141/miR-340 and interacting with YBX1; its knockdown inhibits tumor growth, enhances CD8+T/M1 macrophage infiltration, activates CD8+T via PD-L1, and boosts macrophage phagocytosis through CD47.	TUG1-siRNA combined with PD-L1 antibody can effectively inhibit tumor growth, providing a new target and strategy for HCC immunotherapy.	(51)
LINC01116	LINC01116 inhibits RAD18-mediated ubiquitination, stabilizes EWSR1, upregulates PPARA and FABP1, promotes long-chain fatty acid uptake (e.g., linoleic acid), induces T cell dysfunction, and drives HCC progression.	The LINC01116-EWSR1-PPARA-FABP1 axis may be a new target for cancer immunotherapy.	(54)
CRNDE	CRNDE drives HCC progression by enhancing tumor growth, recruiting G-MDSCs, and inhibiting T cell invasion via TLR3 binding, NF-κB activation, and CXCL3 upregulation.	The CRNDE - NF-κB - CXCL3 axis plays an important role in promoting the immunosuppressive environment and tumor progression in HCC.	(55)
HOTTIP	HOTTIP promotes HCC cell viability by suppressing miR-205; HOTTIP knockdown or miR-205 upregulation inhibits HCC proliferation, while miR-205 downregulation reverses HOTTIP knockdown effects.	LncRNA HOTTIP regulates the carcinogenic mechanism of miR-205 in HCC and provides a potential target for the treatment of HCC.	(57)
MIAT	MIAT and PD-L1 are upregulated in HCC; MIAT promotes STAT3 by inhibiting miR-411-5p, increasing PD-L1 transcription. MIAT knockdown enhances T cell cytotoxicity, while miR-411-5p inhibition reverses STAT3 and PD-L1 reduction.	This study suggests a new lncRNA-mediated immune escape mechanism of HCC cells, and the MIAT/miR-411-5p/STAT3/PD-L1 pathway may be a new therapeutic target for HCC.	(52)
AC099850.3	AC099850.3 promotes tumorigenesis via the PRR11/PI3K/AKT axis, correlates positively with T helper cells and M0 macrophages but negatively with M2 macrophages, monocytes, NK cells, and CD8+ T cells, and is strongly linked to immune checkpoint molecules like PD-1 and PD-L1.	AC099850.3 promotes HCC malignancy and serves as a potential biomarker and therapeutic target for immunotherapy.	(58)
GAS5	lncRNA-GAS5 levels are reduced in PBMC-CD4+ T cells and tumor tissues compared to healthy controls and adjacent non-tumor tissues. Del/Del genotype increases lncRNA-GAS5 expression, lowers postoperative pain, and improves survival. lncRNA-GAS5 negatively correlates with IL-6, IL-17, and Th17 polarization index (RORγT/CD3).	LncRNA-GAS5 expression and its promoter rs145204276 polymorphism are prognostic biomarkers for predicting postoperative pain in patients with hepatectomy.	(59)
XIST	XIST is associated with high tumor and peritumoral CD4 inflammation, higher CD25+ cell counts (p=0.01), and elevated intratumoral CD25 expression.	LncRNA XIST is expressed in CHB-HCC and strongly correlates with inflammatory TME, particularly CD25+ Treg presence and quantity.	(60)
NEAT1	NEAT1 and Tim-3 are upregulated in HCC patients’ PBMCs. NEAT1 downregulation inhibits CD8+T cell apoptosis and enhances cytotoxicity, while miR-155 interference promotes Tim-3 upregulation.	NEAT1 downregulation, via the miR-155/Tim-3 pathway, inhibits CD8+T cell apoptosis and enhances cytotoxicity against HCC, highlighting its potential as an immunotherapy target.	(49)
E2F1	E2F1 positively correlates with memory CD4+T cells, memory B cells, eosinophils, and T helper cells but negatively with monocytes, initial B cells, and resting memory CD4+T cells. It is also positively linked to immune checkpoints (PDCD1, CTLA4, LAG3) and potentially sensitive to 39 drugs.	This study provides an important direction for the study of E2F1, which may contribute to the identification of HCC related molecular biotherapy and immunotherapy targets.	(61)
AIFM2-1	Lnc-AIFM2-1 reduces HBV clearance by CD8+T cells via miR-330-3p inhibition and CD244 accumulation; Lnc-AIFM2-1-siRNA, miR-330-3p mimic, or CD244-siRNA restore CD8+T cell function.	Lnc-AIFM2-1 acts as a ceRNA for miR-330-3p, upregulating CD244 to promote HBV immune escape, revealing lncRNA-miRNA-mRNA interactions in immune evasion. It highlights Lnc-AIFM2-1 and CD244 as potential targets for CHB diagnosis and treatment.	(62)
MIAT	MIAT in HCC negatively correlates with tumor purity but positively with immune cells (B cells, T lymphocytes, macrophages) and checkpoint molecules (PD-1, PD-L1, CTLA4). MIAT-TF interactions regulate genes (JAK2, SLC6A6, KCND1, MEIS3, RIN1) to influence the immune microenvironment and are linked to sensitivity to anticancer drugs, particularly Sorafenib.	This study provides a meaningful attempt to reveal the immune escape mechanism of HCC and find the effectiveness of targeted drugs.	(63)
LINC01132	LINC01132 overexpression drives cell growth, proliferation, invasion, and metastasis by interacting with NRF1 to upregulate DPP4. Silencing LINC01132 induces CD8+ T cell infiltration, enhancing anti-tumor immunity when combined with anti-PD-L1 therapy.	LINC01132 promotes the development of HCC through the NRF1/DPP4 axis, and targeted silencing of LINC01132 is expected to enhance the effect of anti-PD-L1 immunotherapy.	(56)
Lnc-Tim3	Lnc-Tim3 is upregulated in tumor-infiltrating CD8 T cells, inversely correlating with IFN-γ and IL-2 production. It drives CD8 T cell exhaustion and survival by binding to Tim-3, blocking BAT3 interaction, inhibiting Lck/NFAT1/AP-1 signaling, and promoting nuclear Bat3-mediated activation of p300-dependent anti-apoptotic genes (MDM2, Bcl-2).	Lnc-Tim3 drives T cell exhaustion, weakening anti-tumor immunity, and may impact the efficacy of therapies targeting acquired immune system regulation.	(50)
PCED1B-AS1	PCED1B-AS1 inhibits PD-Ls expression by sponging hsa-miR-194-5p, inducing PD-Ls-mediated T cell immunosuppression. HCC exosomes containing PCED1B-AS1 enhance PD-Ls in recipient cells while suppressing T cells and macrophages. Blood exosome PCED1B-AS1 correlates with PD-Ls expression in HCC.	PCED1B-AS1 enhances the expression and function of PD-Ls through spongification of hsa-miR-194-5p and induces immunosuppression of HCC.	(64)
XIST	Somatic CD4+T cell infiltration, especially resting memory CD4+T cell infiltration, was positively correlated with lncRNA and its related mirnas, while Th1 CD4+T cell infiltration was negatively correlated.	These findings provide new insights into the mechanism of immune dysregulation of CD4+T cells in HCC.	(65)
GIHCG	GIHCG is overexpressed in HCC, correlating with poor clinicopathological features, shorter survival, and reduced infiltration of memory CD4+/CD8+ T cells, NK cells, macrophages, dendritic cells, neutrophils, and monocytes.	GIHCG can be used as a biomarker to predict prognosis in HCC patients and is associated with immune cell infiltration in HCC.	(66)
LINC00261	LINC00261, MiR105-5p, and selectin L(SELL) may be potential new biomarkers for patient prognosis and immunotherapy.	The findings provide important insights into the selection of the LINC00261/MiR105-5p/SELL signaling pathway as a novel marker associated with overall survival of HCC and may impact the effectiveness of HCC immunotherapy.	(67)
SNHG3	SNHG3 promotes PD1 expression by regulating ASF1B. Ultimately, elevated ASF1B predicted poor outcomes for HCC patients in subgroups with B cells, CD8+ T cells, or neutropenia, and in subgroups with CD4+ T cell enrichment.	It was found that the novel lncRNA SNHG3/miR-214-3p/ASF1B axis may promote HCC recurrence by regulating immune infiltration.	(68)
FENDRR	FENDRR regulates GADD45B by competitively binding miR-423-5p, which targets GADD45B. FENDRR and GADD45B are downregulated in HCC, while miR-423-5p is upregulated. FENDRR overexpression and miR-423-5p downregulation inhibit HCC proliferation, promote apoptosis, and regulate Treg-mediated immune escape.	miR-423-5p up-regulates GADD45B, thereby inhibiting Treg-mediated immune escape of HCC cells.	(69)
CTD-2510F5.4	CTD-2510F5.4 overexpression promotes HCC proliferation, invasion, and metastasis, correlating with poor prognosis, higher TP53 mutation, increased Treg infiltration, elevated immune checkpoint expression, and impaired immunity. Low CTD-2510F5.4 enhances sensitivity to immunotherapy and anti-angiogenic therapy, while high expression favors chemotherapy response.	CTD-2510F5.4 is crucial in HCC progression and immune regulation, warranting further study as a prognostic biomarker and treatment response predictor to develop personalized HCC therapies.	(53)

#### 3.1.2 Macrophage

Macrophages play a dual role in the immune microenvironment of HCC, capable of both promoting tumor progression and inhibiting tumor growth. LncRNAs regulate macrophage function through various mechanisms, influencing their polarization states, secretory characteristics, and interactions with tumor cells.

Extracellular vesicles (EVs) in the TME play a crucial role in regulating the tumor immune microenvironment. Studies have shown that tumor-associated macrophages (TAMs) secrete exosomes containing the lncRNA MMPA, enhancing aerobic glycolysis and proliferation in HCC cells. Mechanistically, lncMMPA not only promotes M2 macrophage polarization but also acts as a sponge for miR-548, increasing ALDH1A3 mRNA expression and thereby facilitating glucose metabolism and cell proliferation. Clinical data indicate that lncMMPA expression correlates with TAM glycolysis and decreased survival rates in HCC patients (70). Additionally, HCC cell-derived exosomes are found to contain high levels of TUC339, which can be absorbed by macrophages (THP-1 cells). Inhibition of TUC339 enhances the production of pro-inflammatory factors, expression of co-stimulatory molecules, and phagocytic capacity in macrophages, while overexpression of TUC339 suppresses these functions. TUC339 is highly expressed in M2 macrophages and exhibits distinct regulatory patterns during the polarization switch between M1 and M2 macrophages, indicating its key role in M1/M2 polarization. TUC339 is also associated with cytokine receptor interactions, chemokine receptor binding, Toll-like receptor signaling, FcγR-mediated phagocytosis, cytoskeletal regulation, and cell proliferation (71).

Furthermore, the lncRNA FAL1 is highly enriched in exosomes from the serum of HCC patients and promotes tumor progression. Exosomes enriched with FAL1 can induce M2 polarization in macrophages, enhancing the proliferation, invasion, and clonogenicity of HCC cells while inhibiting apoptosis and sensitivity to sorafenib. Interfering with FAL1 can reverse these effects. FAL1 promotes malignant progression in HCC by activating the Wnt/β-catenin signaling pathway (72). Another study reveals the role of the EZH2-related lncRNA HEIH, which is abnormally upregulated in HCC and correlates with poor prognosis in tumor tissues and cell lines. HEIH is enriched in exosomes found in plasma and cell supernatants and is transported to macrophages by HCC-derived exosomes, inducing M2 polarization and promoting HCC cell proliferation, migration, and invasion. Mechanistically, HEIH acts as a miRNA sponge, binding to miR-98-5p, leading to STAT3 upregulation. This provides evidence for the novel regulatory role of tumor-derived exosomal lncRNA HEIH targeting the miR-98-5p/STAT3 axis in macrophages, aiding the understanding of the complex interactions between the TME and immune cells in HCC treatment (73).

HMMR-AS1, an lncRNA associated with poor prognosis, promotes M2 polarization in macrophages by competitively binding to miR-147a, preventing the degradation of ARID3A, and accelerates HCC progression. Additionally, in hypoxic conditions, HIF-1α enhances HMMR-AS1 transcription by binding to its promoter, increasing exosome secretion (74).

Apart from exosomes, lncRNAs can also influence macrophage polarization and function through EVs. The lncRNA PART1 can be transported to macrophages via tumor-derived EVs, regulating macrophage polarization in HCC. PART1 binds to miR-372-3p, inhibiting its expression, while miR-372-3p negatively regulates TLR4 expression in macrophages. EVs containing PART1, TLR4 overexpression, or miR-372-3p inhibition all induce macrophage polarization toward M2. Thus, EVs from HCC cells may promote M2 polarization by transporting PART1 to inhibit miR-372-3p, leading to upregulation of TLR4 (75).

Metabolic reprogramming is also an important pathway through which lncRNAs influence macrophage status. Research has identified that the lncRNA miR4458HG promotes carcinogenesis in HCC, particularly in glucose metabolism. High expression of miR4458HG enhances HCC cell proliferation, activates glycolytic pathways, and induces polarization of TAMs. Mechanistically, miR4458HG stabilizes its target mRNAs (e.g., HK2 and SLC2A1) by binding to IGF2BP2, a key RNA m6A recognition protein, thereby enhancing glycolysis and altering the physiology of tumor cells. Moreover, miR4458HG secreted by HCC can increase ARG1 expression via exosomes, further promoting M2 polarization in macrophages (76).

The aforementioned studies reveal the complex mechanisms by which lncRNAs regulate macrophage polarization and function within the TME, underscoring their significance in the progression of HCC. Notably, lncRNAs such as lncMMPA, TUC339, and FAL1 influence glucose metabolism, inflammatory responses, and cellular signaling pathways, promoting M2 macrophage polarization and thereby creating a favorable environment for the survival and proliferation of HCC cells. These findings not only deepen our understanding of the roles of lncRNAs in the tumor immune microenvironment but also suggest that future therapeutic strategies should consider targeting these specific lncRNAs to reverse macrophage polarization and enhance the efficacy of immunotherapy for HCC. Further research should focus on the interactions between lncRNAs and other components of the TME to comprehensively elucidate their potential mechanisms in tumor progression. Additional relevant studies are summarized in **Table 2**.

**Table 2:** Regulation of Macrophage by lncRNAs in HCC.

LncRNAs	Mechanism and Effect	Clinical significance	Ref
Cox-2	lncRNA Cox-2 is highly expressed in M1 macrophages; its knockdown reduces pro-inflammatory factors (IL-12, iNOS, TNF-α) in M1 and increases anti-inflammatory factors (IL-10, Arg-1, Fizz-1) in M2. It also weakens M1’s ability to inhibit HCC proliferation, invasion, and migration while enhancing M2’s pro-tumor effects.	LncRNA Cox-2 inhibits immune escape and tumor growth of HCC by inhibiting the polarization of M2-type macrophages.	(77)
MMPA	TAMs promote HCC aerobic glycolysis and proliferation via exosomal lncMMPA, which polarizes M2 macrophages and upregulates ALDH1A3 mRNA by sponging miR-548, enhancing glucose metabolism and cell growth.	LncMMPA plays an important role in malignant transformation and metabolic reprogramming of HCC by regulating miR-548/ALDH1A3 pathway.	(70)
TUC339	HCC exosomes containing TUC339 are absorbed by macrophages; TUC339 inhibition enhances proinflammatory cytokine production, co-stimulatory molecule expression, and phagocytosis, while its overexpression suppresses these functions. TUC339 is highly expressed in M2 macrophages and regulates M1/M2 polarization, impacting cytokine receptor interactions, chemokine binding, toll-like receptor signaling, phagocytosis, cytoskeletal regulation, and cell proliferation.	Tumor-derived exosomal lncRNA TUC339 regulates macrophage behavior, revealing a key mechanism of tumor-immune cell communication via exosomal lncRNAs.	(71)
ELMO1-AS1	High levels of ELMO1-AS1 are associated with better treatment outcomes and may be an independent prognostic marker for HCC. Overexpression of ELMO1-AS1 can inhibit the proliferation, migration and invasion of HCC cells, and its target may be ELMO1.	ELMO1-AS1 may be an important regulator of liver cancer progression and has potential as a prognostic marker and therapeutic target.	(78)
HMMR-AS1	HMMR-AS1 is upregulated in HCC, correlating with poor prognosis; its inhibition suppresses tumor growth. It promotes M2 macrophage polarization and HCC progression by competitively binding miR-147a to stabilize ARID3A. HIF-1α enhances HMMR-AS1 transcription and exosome secretion under hypoxia.	These findings provide new ideas for the study of HCC pathogenesis and the development of targeted therapies.	(74)
FAL1	FAL1-enriched exosomes induce M2 macrophage polarization, enhancing HCC proliferation, invasion, and clonal formation while reducing apoptosis and sorafenib sensitivity. FAL1 interference reverses these effects by inhibiting the Wnt/β-catenin pathway. FAL1-enriched exosomal macrophages also promote tumor growth in mouse models.	FAL1 in exosomes promotes HCC progression by inducing M2-type polarization in macrophages and activating the Wnt/beta-catenin signaling pathway.	(72)
MEG3	MEG3 overexpression promotes M1 macrophage polarization, increases Th1 cytokines, and reduces CSF-1 and PD-1/PD-Ls, inhibiting HCC growth, invasion, and migration. MEG3 knockdown (KD) induces M2 polarization, elevates Th2 cytokines, CSF-1, and PD-1/PD-Ls, and promotes tumor growth. *In vivo*, MEG3 OE inhibits tumor growth and PD-1/PD-Ls while enhancing Th1 immunity, whereas MEG3 KD has the opposite effect.	MEG3 regulates the TME of HCC by influencing macrophage polarization and T cell immune balance, and becomes a potential therapeutic target.	(79)
miR4458HG	miR4458HG promotes HCC proliferation, activates glycolysis, and induces TAM polarization by stabilizing target mRNAs (e.g., HK2, SLC2A1) via IGF2BP2 binding. HCC-secreted miR4458HG exosomes increase ARG1 expression, further driving M2 macrophage polarization.	MiR4458HG is considered to be a pro-cancer factor in HCC, and targeting this pathway may provide new strategies for HCC patients with high glucose metabolism.	(76)
HEIH	HCC exosomes deliver HEIH to macrophages, inducing M2 polarization and promoting HCC proliferation, migration, and invasion. HEIH acts as a miR-98-5p sponge, upregulating STAT3, as confirmed in tumor xenograft models.	This study reveals the novel role of tumor-derived exosomal lncRNA HEIH in regulating macrophages via the miR-98-5p/STAT3 axis, advancing understanding of TME-immune cell interactions in HCC therapy.	(73)
MEG3	MEG3 promotes M1 polarization and inhibits M2 polarization of BMDM by binding HuR, which suppresses CCL5 transcription. MEG3 overexpression inhibits HCC metastasis, invasion, and angiogenesis by blocking M2 polarization. MEG3 suppresses tumorigenesis by enhancing M1 and inhibiting M2 polarization, while CCL5 absence in M2 macrophages reverses MEG3’s inhibitory effects on migration, invasion, and lumen formation.	MEG3 inhibits HCC progression by binding to HuR and upregulating CCL5.	(80)
MAAS	HBeAg secreted by HBV+HCC cells up-regulates MAAS expression in M2 macrophages by influencing m6A modification of MAAS, and then transfers to HBV+HCC cells via exosomes to promote their proliferation and target c-Myc	/	(81)
MEG3	MEG3 expression rises during LPS/IFN-γ-induced M1 polarization but declines during IL4/IL13-induced M2 polarization. MEG3 overexpression suppresses M2 markers *in vitro* and *in vivo* by binding miR-145-5p to upregulate DAB2, inhibiting M2-induced HCC metastasis, angiogenesis, and tumor growth.	LncRNA MEG3 inhibits the polarization of M2 macrophages through the miR-145-5p/DAB2 axis, thereby inhibiting the development of HCC.	(82)
CRNDE	Conditioned medium (CM) of M2 cells and CRNDE overexpressed M0 macrophages promoted HCC cell survival, migration, and invasion, while CRNDE knockdown of M0 cell CM reversed this effect. CRNDE overexpressed M0 cells promoted tumor growth and the expression of Ki67 and CD206.	CRNDE regulates metabolic reprogramming of M2 macrophages through the ERK pathway, thereby promoting the proliferation, migration and invasion of HCC.	(83)
TP73-AS1	TP73-AS1 negatively regulates miR-539, and its knockdown inhibits MMP-8 expression and M2 macrophage polarization. miR-539 overexpression also suppresses M2 polarization by downregulating MMP-8. MMP-8 knockdown limits M2 polarization by inhibiting TGF-β1 signaling. TP73-AS1 knockdown or miR-539 overexpression inhibits HCC tumor growth and M2 macrophage infiltration.	LncRNA TP73-AS1 promotes the expression of MMP-8 by negatively regulating miR-539, and then activates TGF-β1 signaling pathway to induce polarization of M2 macrophages in HCC.	(84)
MA301	Lnc-MA301 is upregulated during M2-to-M1 polarization of U937 macrophages. In HCC, its expression is lower than in adjacent tissues and correlates with poor prognosis. Lnc-MA301 activation inhibits HCC proliferation, migration, EMT, and lung metastasis in mice by interacting with caprin-1 via the Akt/Erk1 pathway.	Lnc-MA301 may play a regulatory role in the development and metastasis of HCC.	(85)
PART1	PART1 and TLR4 are upregulated, while miR-372-3p is downregulated in HCC. PART1 promotes HCC proliferation, migration, invasion, and EMT by binding miR-372-3p, which negatively regulates TLR4. PART1-containing EVs or miR-372-3p inhibition induces M2 macrophage polarization. PART1 EVs drive HCC progression and M2 polarization via the miR-372-3p/TLR4 axis.	HCC cell-derived EVs may inhibit miR-372-3p through PART1 transport, thereby up-regulating TLR4 and promoting M2 polarization of macrophages in HCC.	(75)
LINC00662	LINC00662 activates Wnt/β-catenin signaling in macrophages via WNT3A secretion, driving M2 polarization. This promotes HCC tumor growth and metastasis by enhancing Wnt/β-catenin signaling and M2 macrophage polarization.	Targeting LINC00662 may provide a new therapeutic strategy for HCC.	(86)
MALAT1	MALAT1 knockdown reduces VEGF-A production, impairs HUVEC angiogenesis, and promotes M1 macrophage polarization. MALAT1 interacts with miR-140, which targets VEGF-A, and their levels are negatively correlated in HCC. miR-140 inhibition increases VEGF-A, enhances HUVEC angiogenesis, and drives M2 polarization.	This study reveals the mechanism by which MALAT1 acts as a promoter of HCC, possibly by inhibiting miR-140. Therefore, targeting MALAT1 or miR-140 may help slow the progression of HCC.	(87)

#### 3.1.3 Others

In addition to T cells and macrophages, other cell types in the tumor immune microenvironment are also regulated by lncrnas. Previous studies have shown that a variety of lncRNAs are associated with tumor invasion of B cells. For example, lncRNA AC099850.3 and E2F1 are positively correlated with the level of memory B cells (58, 61), while RNASEH1-AS1 is negatively correlated with the invasion of B cells (88). NK, as an immune cell responsible for recognizing and killing tumor cells, can also be regulated by LncRNAs through a variety of mechanisms.For example, LINC00707 can interact with YTHDF2, promoting ubiquitination dependent degradation of YTHDF2 protein. It can also influence the cytotoxicity of NK-92MI cells to HCC cells through its interaction with YTHDF2 (89). Treg is an immunosuppressive cell. Previous studies have shown that lncRNA FENDRR up-regulates GADD45B by adsorbing miR-423-5p, and inhibits Treg-mediated immune escape of HCC cells (69).

The previous sections primarily focused on immune-suppressive lncRNAs; however, in addition to those lncRNAs that promote tumor growth and immune suppression, several lncRNAs have been identified as tumor suppressors, playing critical roles in enhancing immune responses and improving survival outcomes in HCC. For example, MEG3 expression increases during LPS/IFNγ-induced M1 polarization, while it decreases during IL4/IL13-induced M2 polarization. Overexpression of MEG3 suppresses M2 polarization markers in mice. Mechanistically, MEG3 regulates DAB2 expression by binding to miR-145-5p. Overexpressing MEG3 inhibits HCC cell metastasis and angiogenesis induced by M2 polarization, and it suppresses tumor growth *in vivo* (82). Knockdown of MALAT1 inhibited VEGF-A production, impaired the angiogenesis of HUVECs, and promoted the polarization of macrophages to the M1 subgroup. Mechanistic studies confirmed the interaction between MALAT1 and miR-140 and between miR-140 and VEGF-A, revealing a negative correlation between MALAT1 and miR-140 in HCC tissues. In addition, inhibition of miR-140 significantly increased VEGF-A expression, promoted angiogenesis in HUVECs, and polarized macrophages toward the M2 subpopulation (87). LINC00261 has been shown to act as a tumor suppressor in various cancers. Studies indicate that in HCC, LINC00261 expression is downregulated in tumor tissues and is associated with better prognosis. Furthermore, the LINC00261/MiR105-5p/SELL axis is involved in B-cell dysfunction. These findings provide significant insights into selecting the LINC00261/MiR105-5p/SELL signaling pathway as a new biomarker related to overall survival in HCC and may influence the effectiveness of HCC immunotherapy (67). Previous research has shown that JPX-induced XIST inhibits liver cancer progression by sequestering miR-155-5p (90). XIST is more highly expressed in patients with high tumor and peritumoral CD4 inflammation. The number of CD25-positive cells is significantly higher in tumors with XIST expression, suggesting its potential regulatory role in the immune microenvironment (60). It is worth noting that the clinical application of these lncRNAs still faces significant challenges and requires further clinical validation. These relevant lncRNAs are summarized in the corresponding table according to the above classification.

### 3.2 LncRNAs in immune checkpoint regulation

In the TME, lncRNAs play a crucial role in immune regulation by modulating the expression of PD1 and its ligand PD-L1 (**Figure 2B**). These lncRNAs are involved in the mechanisms of immune evasion and influence the signaling between tumor cells and immune cells through interactions with miRNAs or transcription factors. Research indicates that the upregulation of certain lncRNAs is closely associated with the expression of PD1/PD-L1, thereby promoting the survival and proliferation of tumor cells.

Previous studies have indicated that various lncRNAs are positively correlated with the expression of PD1/PD-L1 or can promote their expression. For example, AC099850.3 is significantly positively correlated with key immune checkpoint molecules (PD1, PD-L1, PD-L2, and CTLA4) (58). MIAT promotes the expression of PD-L1 at the transcriptional level by downregulating miR-411-5p, which in turn enhances STAT3 expression (52). SNHG3 can also promote PD1 expression by regulating ASF1B (68). Hypoxia induces the interaction between MIR155HG and ILF3, stabilizing HIF-1α mRNA and increasing PD-L1 expression, thereby facilitating immune evasion in HCC (91). Additionally, MIR155HG functions as a competitive endogenous RNA (ceRNA) that regulates PD-L1 expression through the miR-223/STAT1 axis (92). HOXA-AS3 is highly expressed in HCC cells and enhances PD-L1 expression by adsorbing miR-455-5p, thereby increasing the proliferation, migration, and invasion abilities of HCC cells, regulating the cell cycle, and inhibiting apoptosis (93).

Multiple mechanisms are involved in the regulation of PD1/PD-L1 by lncRNAs. PCED1B-AS1 enhances the expression of PD-L1 and PD-L2 by adsorbing hsa-miR-194-5p, thereby increasing the immune suppressive ability of HCC cells. Furthermore, HCC cells release exosomes containing PCED1B-AS1, transmitting this immune suppressive mechanism to other HCC cells, T cells, and macrophages. High expression of PCED1B-AS1 is closely related to the overexpression of PD-Ls in HCC and promotes tumor cell proliferation and tumor formation while inhibiting apoptosis (64). METTL3-mediated m6A modification upregulates TUG1, promoting tumor immune evasion. Silencing TUG1 inhibits tumor growth and metastasis, enhances the infiltration of CD8+ T cells and M1 macrophages, and activates CD8+ T cells through PD-L1 while promoting macrophage phagocytosis via CD47. TUG1 regulates the expression of PD-L1 and CD47 by adsorbing miR-141 and miR-340, respectively, and interacts with YBX1 to promote their transcriptional upregulation, ultimately facilitating tumor immune evasion (51).

In addition to PD1/PD-L1, lncRNAs are also associated with other checkpoints such as CTLA-4 and TIM-3, and they can even regulate their levels and functions. For example, Lnc-Tim3 specifically binds to Tim-3, obstructing its interaction with Bat3, which inhibits the downstream Lck/NFAT1/AP-1 signaling pathway, leading to the nuclear localization of Bat3 and enhancing the transcriptional activity of p300-dependent p53 and RelA. This promotes the transcriptional activation of anti-apoptotic genes (such as MDM2 and Bcl-2), contributing to T cell exhaustion, which is linked to diminished anti-tumor immunity (50). Furthermore, a study established a prognostic model for HCC composed of 12 ferroptosis-related lncRNAs, dividing HCC patients into high-risk and low-risk groups. In the high-risk group, the expression of various immune checkpoint molecules, including PD1, CTLA-4, and CD86, was significantly elevated (94). Another study constructed a new feature comprising three ferroptosis-related lncRNAs, which was associated with immune cell infiltration (such as macrophages, myeloid dendritic cells, and neutrophils) and immune checkpoint blockade targets (including PD1, CTLA-4, and TIM-3) (95).

The above studies clearly demonstrate the significant role of lncRNAs in regulating immune checkpoints in HCC. We have summarized the related research in **Table 3** for a more intuitive presentation.

**Table 3:** Regulation of Immune Checkpoint by lncRNAs in HCC.

LncRNAs	Mechanism and Effect	Clinical significance	Ref
MIAT	MIAT and PD-L1 are upregulated in HCC. MIAT promotes STAT3 by downregulating miR-411-5p, increasing PD-L1 transcription. MIAT knockdown enhances T cell cytotoxicity, and miR-411-5p inhibition reverses STAT3 and PD-L1 suppression.	This study reveals the MIAT-mediated mechanism of immune evasion in HCC, suggesting that the MIAT/miR-411-5p/STAT3/PD-L1 axis could serve as a novel therapeutic target for HCC treatment.	(52)
MIR155HG	HIF-1α binds to the MIR155HG promoter, enhancing its transcription. Hypoxia promotes ILF3 nuclear export, increasing ILF3-MIR155HG interaction, stabilizing HIF-1α mRNA, and elevating PD-L1 expression.	This study reveals that hypoxia promotes HCC immune evasion by inducing the interaction between MIR155HG and ILF3, which stabilizes HIF-1α mRNA and enhances PD-L1 expression.	(91)
PCED1B-AS1	PCED1B-AS1 enhances HCC immunosuppression by sponging hsa-miR-194-5p, increasing PD-L1 and PD-L2 expression. It blocks hsa-miR-194-5p’s inhibition of PD-Ls, leading to their overexpression. HCC exosomes containing PCED1B-AS1 spread this mechanism to other HCC cells, T cells, and macrophages.	PCED1B-AS1 induces immune evasion in HCC by regulating the expression of PD-Ls, suggesting it may serve as a novel therapeutic target for HCC treatment.	(64)
HOXA-AS3	HOXA-AS3 is highly expressed in HCC cells and promotes PD-L1 expression by sequestering miR-455-5p, thereby enhancing HCC cell proliferation, migration, and invasion, regulating the cell cycle, and inhibiting apoptosis.	HOXA-AS3 exerts its effects in HCC by regulating the miR-455-5p/PD-L1 axis, suggesting it may serve as a new therapeutic target for HCC treatment.	(93)
TUG1	TUG1, upregulated by METTL3-mediated m6A modification, promotes immune evasion. TUG1 knockdown inhibits tumor growth and metastasis, enhances CD8+ T cell and M1 macrophage infiltration, activates CD8+ T cells via PD-L1, and promotes macrophage phagocytosis via CD47. TUG1 sequesters miR-141 and miR-340 to regulate PD-L1 and CD47, respectively, and interacts with YBX1 to upregulate their transcription, facilitating immune escape.	This mechanism provides new insights for enhancing immune therapy in HCC.	(51)
MIR155HG	LPS induces PD-1 and PD-L1 expression, significantly upregulating PD-L1 in HCC. LPS enhances METTL14, promoting m6A methylation of MIR155HG, which is stabilized by ELAVL1. MIR155HG acts as a ceRNA, regulating PD-L1 via the miR-223/STAT1 axis.	This mechanism provides new therapeutic targets for the immunotherapy of HCC, particularly in cases associated with liver cirrhosis.	(92)
AC099850.3	AC099850.3 is significantly positively correlated with immune checkpoint molecules (PD-1, PD-L1, PD-L2, and CTLA4).	This indicates its potential as a therapeutic target for immunotherapy in HCC.	(58)
LINC00244	Long non-coding RNA LINC00244 can downregulate the expression of PD-L1, thereby inhibiting the proliferation, invasion, and metastasis of HCC.	High expression of LINC00244 is associated with better clinical prognosis, suggesting that it may be a potential therapeutic target for HCC.	(96)
KCNQ1OT1	KCNQ1OT1, as a ceRNA for miR-506, elevates PD-L1 expression in sorafenib-resistant HCC cells.	KCNQ1OT1 promotes PD-L1-mediated immune evasion and resistance in sorafenib-resistant HCC cells by inhibiting miR-506.	(97)
FOXD1-AS1	FOXD1-AS1, upregulated in malignancies, correlates with poor prognosis and enhances HCC immune evasion by increasing PD-L1 as a ceRNA for miR-615-3p. It also regulates CTC EMT via PI3K/AKT signaling.	This study provides strong evidence supporting the role of FOXD1-AS1 as a miRNA sponge, sequestering miR-615-3p and protecting PD-L1 from inhibition.	(98)
SNHG3	SNHG3 promotes the expression of PD1 by regulating ASF1B.	The study identified a novel lncRNA SNHG3/miR-214-3p/ASF1B axis that promotes the recurrence of HCC by modulating immune infiltration.	(68)
Lnc-Tim3	Lnc-Tim3 binds Tim-3, blocking its interaction with Bat3 and suppressing the Lnc/NFAT1/AP-1 pathway. This drives Bat3 nuclear localization, enhancing p300-dependent p53 and RelA transcriptional activity, activating anti-apoptotic genes (e.g., MDM2, Bcl-2), and promoting T cell exhaustion.	This suggests that Lnc-Tim3 and its associated signaling pathways may influence the efficacy of cancer therapies aimed at modulating the adaptive immune system.	(50)
NEAT1	In the PBMCs of HCC patients, the expression levels of NEAT1 and Tim-3 are significantly elevated. Downregulation of NEAT1 inhibits CD8+ T cell apoptosis and enhances their cytolytic activity, while interference with miR-155 shows the opposite effect by upregulating Tim-3.	NEAT1 may serve as an effective target for enhancing the efficacy of immunotherapy.	(49)

### 3.3 Regulatory roles of LncRNA in cytokines and immune modulators

Gaining a deeper understanding of the role of lncRNAs in cytokines and immune regulatory molecules is also a crucial aspect of providing new strategies and targets for tumor immunotherapy.

TGF-β plays a dual role in HCC. In the early stages, it inhibits cell proliferation and induces apoptosis, serving an anti-tumor role. However, in later stages of HCC, TGF-β promotes tumor cell migration, invasion, and fibrosis in the TME, thereby facilitating tumor progression. Therefore, TGF-β is an important target in HCC treatment. Previous studies have shown that lncRNAs can interact with TGF-β. Research indicates that some lncRNAs can be activated by TGF-β-related pathways in HCC, thereby exerting downstream effects. For instance, lnc-UTGF is transcriptionally induced by TGF-β. Under TGF-β stimulation, SMAD2/3 bind to the lnc-UTGF promoter and activate its expression. Overexpression of lnc-UTGF enhances TGF-β/SMAD signaling, while its knockdown suppresses this signaling. lnc-UTGF interacts with the mRNAs of SMAD2 and SMAD4 through complementary base pairing, thereby increasing the stability of SMAD2/4 mRNA. This indicates the existence of a novel TGF-β/SMAD/lnc-UTGF positive feedback loop (99). Additionally, LINC01980 promotes HCC metastasis via the miR-376b-5p/E2F5 axis under classical TGF-β/SMAD pathway activation (100). TGF-β-activated lncRNA-ATB is upregulated in HCC metastasis and is associated with poor prognosis. lncRNA-ATB induces epithelial-mesenchymal transition (EMT) and cell invasion by competitively binding to the miR-200 family, leading to the upregulation of ZEB1 and ZEB2. Furthermore, lncRNA-ATB promotes the autocrine induction of IL-11 by binding to IL-11 mRNA, activating the STAT3 signaling pathway, and further facilitating the organ colonization of disseminated tumor cells (101). Other studies have shown that some lncRNAs can exert effects by regulating TGF-β secretion. For example, PRR34-AS1 stabilizes the mRNA level of the exosomal protein Rab27a by recruiting DDX3X, thereby promoting the exosomal secretion of VEGF and TGF-β in HCC cells (102). CASC7 can also promote HCC progression by binding to miR-30a-5p, regulating KLF10 and its downstream TGF-β/SMAD3 pathway (103). The downregulation of LINC01278 can reduce HCC cell migration and invasion induced by β-catenin and TGF-β1 (104). HANR and miR-214 jointly regulate EZH2, affecting the level of TGFBR2 (105). Additionally, some lncRNAs participate in TGF-β-induced biological processes. For instance, lncRNA SLC7A11-AS1 and hsa_circ_0006123 are involved in the TGF-β-induced EMT process and may promote HCC metastasis (106).

In addition to TGF-β, lncRNAs also interact with various cytokines and immune regulatory factors such as IL-6, IL-10, INF-γ, and TNF-α (**Figure 2C**). For instance, lnc-DILC exerts its effects by inhibiting the autocrine IL-6/STAT3 signaling pathway, and there are binding sites between lnc-DILC and the IL-6 promoter. Furthermore, lnc-DILC mediates the interaction between TNF-α/NF-κB signaling and IL-6/STAT3 signaling (107). Both TNF-α and IL-6 can also regulate the expression of lncRNA 00607 (108). The downregulation of lncRNA cox-2 using siRNA significantly decreased the expression of IL-12, iNOS, and TNF-α in M1 macrophages, while increasing the expression of IL-10, Arg-1, and Fizz-1 in M2 macrophages (77). Relevant studies are summarized in **Table 4** for a more intuitive presentation.

**Table 4:** Regulation of Cytokines and Immune Modulators by lncRNAs in HCC.

LncRNAs	Mechanism and Effect	Clinical significance	Ref
LINC01980	Under the activation of the classical TGF-β/SMAD pathway, LINC01980 promotes HCC metastasis through the miR-376b-5p/E2F5 axis.	LINC01980 may serve as a potential prognostic biomarker and therapeutic target for HCC.	(100)
ATB	TGF-β-activated lncRNA-ATB is upregulated in HCC metastasis and linked to poor prognosis. It induces EMT and invasion by sponging miR-200 family, upregulating ZEB1 and ZEB2. lncRNA-ATB also binds IL-11 mRNA, promoting autocrine IL-11 induction and STAT3 activation, enhancing tumor cell colonization.	lncRNA-ATB, a TGF-β signaling mediator, increases HCC metastasis risk and represents a potential anti-metastatic therapy target.	(101)
LINC01278	The downregulation of LINC01278 reduces the migration and invasion of HCC cells induced by β-catenin and TGF-β1.	The study uncovers the β-catenin/TCF-4-LINC01278-miR-1258-Smad2/3 feedback loop in HCC metastasis, highlighting LINC01278 as a potential therapeutic target.	(104)
MIR4435-2HG	HSC-released CXCL1 exacerbates the malignant behavior of HCC cells through the MIR4435-2HG/miR-506-3p/TGFB1 axis.	CXCL1 and the MIR4435-2HG/miR-506-3p/TGFB1 axis may also represent potential therapeutic targets for HCC treatment.	(109)
SLC7A11-AS	LncRNA SLC7A11-AS1 and hsa_circ_0006123 are involved in the TGF-β-induced EMT process and may promote the metastasis of HCC.	These findings advance clinical diagnostic and therapeutic approaches, offering new avenues for research into TGF-β-induced EMT molecular mechanisms.	(106)
HANR	Both HANR and miR-214 jointly regulate EZH2, thereby affecting the levels of TGFBR2.	The study reveals the HANR/miR-214/EZH2/TGF-β axis as a novel regulatory mechanism in HCC tumorigenesis and progression, providing insights for therapeutic strategies and diagnostic biomarkers targeting HANR.	(105)
UTGF	Lnc-UTGF is induced by TGF-β via SMAD2/3 binding to its promoter. Its overexpression enhances TGF-β/SMAD signaling, while knockdown inhibits it. Lnc-UTGF stabilizes SMAD2/4 mRNAs through base pairing, forming a TGF-β/SMAD/lnc-UTGF positive feedback loop.	The lnc-UTGF-mediated positive feedback loop in TGF-β signaling, coupled with its role in liver cancer metastasis, highlights its potential as a therapeutic target for HCC metastasis.	(99)
CASC7	CASC7 regulates KLF10 and its downstream TGF-β/SMAD3 pathway by binding to miR-30a-5p, thereby promoting the progression of HCC cells.	CASC7 is involved in the tumorigenesis and progression of HCC by regulating miR-30a-5p and its target KLF10.	(103)
NNT-AS1	Inhibition of NNT-AS1 significantly reduces the levels of TGF-β, TGFBR1, and SMAD5 in HCC cells.	NNT-AS1 activates the TGF-β signaling pathway, inhibiting CD4+ T cell infiltration and offering new insights into HCC immune evasion.	(110)
AK021443	Overexpression of AK021443 significantly increases the release of inflammatory factors such as TGF-β, interleukin-1β, platelet-derived growth factor, epidermal growth factor, and ROS.	Overexpression of AK021443 enhances the proliferation, activation, and pro-inflammatory response of HSCs, suggesting its potential significant role in exacerbating liver fibrosis.	(111)
PRR34-AS1	PRR34-AS1 stabilizes the mRNA levels of the exosomal protein Rab27a by recruiting DDX3X, thereby promoting the secretion of VEGF and TGF-β in HCC cells.	This study uncovers PRR34-AS1’s role in enhancing exosomal secretion in HCC cells, offering new insights into lncRNA-mediated regulation of tumor cell biology.	(102)
LINC00974	LINC00974 acts as a ceRNA for miR-642, upregulating KRT19. Its elevation results from promoter hypomethylation, activating Notch and TGF-β signaling pathways.	The LINC00974-KRT19 interaction may serve as a novel diagnostic marker for HCC growth and metastasis, while LINC00974 itself could be a therapeutic target to prevent HCC progression.	(112)
DANCR	DANCR activates the IL-6/STAT3 signaling pathway through PSMD10. The study also found that the activation of the IL-6/STAT3 signaling pathway enhances the transcription of DANCR, creating a positive feedback loop.	This study reveals DANCR’s role in sorafenib resistance via the PSMD10-IL-6/STAT3 axis, offering new therapeutic targets for HCC.	(113)
DLGAP1-AS1	DLGAP1-AS1 sponges miR-26a/b-5p, elevating IL-6 and activating JAK2/STAT3 signaling, which enhances DLGAP1-AS1 transcription, forming a positive feedback loop.	The study reveals DLGAP1-AS1’s role in HCC progression and EMT, highlighting its potential as a therapeutic target.	(114)
MALAT1	MALAT1 promotes the binding of NF-κB to the promoter regions of IL-6 and CXCL8 by recruiting the catalytic subunit BRG1 of the chromatin remodeling complex SWI/SNF, thereby inducing the expression of these inflammatory factors.	Inhibiting MALAT1 may represent a novel strategy for the treatment of HCC.	(115)
TPTEP1	TPTEP1 interacts with STAT3 to inhibit its phosphorylation, homodimerization, nuclear transport, and the transcription of downstream genes.	TPTEP1 suppresses the progression of HCC cells by affecting the IL-6/STAT3 signaling pathway, revealing its tumor-suppressive role in HCC chemotherapy.	(116)
DILC	Lnc-DILC inhibits IL-6/STAT3 signaling by binding to the IL-6 promoter and mediates crosstalk between TNF-α/NF-κB and IL-6/STAT3 pathways.	Lnc-DILC may not only serve as a prognostic biomarker for HCC but also as a potential therapeutic target against liver cancer stem cells.	(107)
Cox-2	The downregulation of lncRNA cox-2 using siRNA significantly reduced the expression of IL-12, iNOS, and TNF-α in M1 macrophages, while increasing the expression of IL-10, Arg-1, and Fizz-1 in M2 macrophages.	lncRNA cox-2 inhibits the polarization of M2 macrophages, thereby suppressing immune evasion and tumor growth in HCC.	(77)
Lnc-00607	TNF-α and IL-6 can regulate the expression of lncRNA 00607. lncRNA 00607 inhibits the transcription of NF-κB p65 by binding to its promoter region, thereby promoting the elevation of p53 levels in HCC.	The TNF-α/IL-6-lncRNA 00607-NF-κB p65/p53 signaling pathway may offer a novel therapeutic strategy for cancer chemotherapy.	(108)
PRRT3-AS1	PRRT3-AS1 knockdown increases IL-10 and TGF-β1 (anti-inflammatory) and decreases IL-1β, TNF-α, and IL-6 (pro-inflammatory) in the cell supernatant.	This has significant clinical implications for predicting patient prognosis and guiding personalized treatment for HCC patients, although further prospective validation is needed.	(117)
TUC339	Inhibiting TUC339 in macrophages reduces the expression of M(IL-4) markers after IL-4 treatment, while overexpressing TUC339 following IFN-γ + LPS treatment enhances the expression of M(IL-4) markers.	The study highlights tumor-derived exosomal lncRNA TUC339’s role in regulating environmental macrophages, uncovering complex tumor-immune cell interactions mediated by exosomal lncRNAs.	(71)
Lnc-Tim3	Lnc-Tim3 is upregulated in tumor-infiltrating CD8 T cells of HCC patients and is negatively correlated with the production of IFN-γ and IL-2.	Lnc-Tim3 and its associated signaling pathways may influence the efficacy of cancer treatments targeting the regulatory adaptive immune system.	(50)

## 4 Potential of lncRNAs in HCC immunotherapy

### 4.1 lncRNAs as biomarkers in HCC immunotherapy

LncRNAs have emerged as promising biomarkers for predicting responses to immunotherapy in HCC. Their unique expression profiles in tumor tissues and circulation provide valuable insights into the TME and immune landscape.

In the previous sections, we discussed specific lncRNAs that are associated with immune cell infiltration, cytokine profiles, and overall immune responses, making them potential prognostic indicators for HCC patients undergoing immunotherapy. For example, lnc-TUG1, lnc-MMPA, lnc-MIAT, and lnc-ATB are lncRNAs whose expression levels are correlated with the state of the tumor immune microenvironment in HCC, suggesting their roles in regulating immune responses (51, 52, 70, 101).

Furthermore, the expression levels of lncRNAs can serve as predictive markers for patient outcomes. By assessing lncRNA profiles, clinicians may better stratify patients, identifying those who are more likely to benefit from immunotherapeutic approaches. This stratification could lead to more personalized treatment strategies, maximizing efficacy while minimizing unnecessary exposure to ineffective therapies. Previous studies have constructed various risk prediction models composed of lncRNAs. For example, one study developed a nine-lncRNA ferroptosis-related signature (CTD-2033A16.3, CTD-2116N20.1, CTD-2510F5.4, DDX11-AS1, LINC00942, LINC01224, LINC01231, LINC01508, and ZFPM2-AS1) to predict the prognosis and immune response in liver cancer. This lncRNA signature may regulate the immune microenvironment of HCC by interfering with TNF-α/NF-κB, IL-2/STAT5 and cytokine/receptor signaling pathways (118). Other studies have also constructed prognostic models using nine copper nephropathy-related lncRNAs. The low-risk group had significantly higher infiltration of immune cells, which primarily have anti-tumor immune functions, and showed a higher sensitivity to ICIs therapy, possibly acting through the AL365361.1/hsa-miR-17-5p/NLRP3 axis. In addition, NLRP3 mutation-sensitive drugs such as VNLG/124, Sunitinib, and rifampicin may achieve better clinical outcomes in high-risk patients (119). More relevant models are summarized in **Table 5**.

**Table 5:** Various risk prediction models composed of lncRNAs in HCC.

LncRNAs	Mechanism and Effect	Clinical significance	Ref
CTD-2033A16.3, CTD-2116N20.1, CTD-2510F5.4, DDX11-AS1, LINC00942, LINC01224, LINC01231, LINC01508, and ZFPM2-AS1	This lncRNA signature may modulate the HCC immune microenvironment by affecting TNF-α/NF-κB, IL-2/STAT5, and cytokine/receptor signaling pathways.	It provides a personalized prediction tool for prognosis and immune response of HCC patients.	(118)
AC073611.1, AL050341.2, LINC02321, LUCAT1, LINC02362, LINC01871, ZNF582-AS	High ATLS scores correlate with poor prognosis, increased tumor mutations, immune activation, elevated T cell proliferation regulators, anti-PD-L1 response, and abnormal sensitivity to oxaliplatin/fluorouracil/Lenvatinib.	ATLS can be used as a powerful biomarker to improve the clinical prognosis and precision treatment of HCC.	(120)
LUCAT1, AC010761.1, AC006504.7 and MIR210HG	MIR210HG silencing inhibits HCC growth and migration by up-regulating PFKFB4 and SPAG4.	This angiogenesis-associated risk model serves as a reliable and promising tool for predicting HCC prognosis.	(121)
MIR4435-2HG/hsa-miR-1-3p/MMP9/hsa-miR-29-3p/DUXAP8	The MIR4435-2HG/hsa-miR-1-3p/MMP9/hsa-miR-29-3p/DUXAP8 ceRNA network correlates with tumor stage and invasion depth. MMP9 positively associates with resting M0 macrophages and NK cells but negatively with activated/resting mast cells, monocytes, and activated NK cells. DUXAP8 positively links to M2 macrophages but negatively to MIR4435-2HG, which itself negatively associates with activated mast cells, CD8+ T cells, and follicular helper T cells.	These findings enhance understanding of immune-related gene interactions with non-coding RNAs in HCC development and progression. Relevant RNAs may serve as diagnostic/prognostic biomarkers and molecular targets for HCC patients.	(122)
GSEC/miR-101-3p/SNX16/PAPOLG	The GSEC/miR-101-3p/SNX16/PAPOLG axis is linked to HCC prognosis. GSEC, SNX16, and PAPOLG are highly expressed in HCC with poor prognosis, while miR-101-3p is low with good prognosis. This axis may influence macrophage polarization.	The GSEC/miR-101-3p/SNX16/PAPOLG axis may be an important factor related to HCC prognosis and immune infiltration.	(123)
LINC01134, C2orf27A, LINC00501, AC104066.3, AC034229.4, CASC8, FAM225B, AL451069.3, AL161669.3, AC116025.2 and LINC00632.	Among the 11-LNCPS lncRNAs, LINC01134 and AC116025.2 are particularly critical. Their upregulation impacts immune cell infiltration, correlates with TCE, poorer OS, and reduced immune response in liver cancer.	LncRNAs in 11-LNCPS influence cancer-related biological processes and signaling pathways, particularly in immune function and metabolism. 11-LNCPS can predict HCC prognosis and immune response.	(124)
AC012073.1, AL031985.3, AL355574.1, LINC01224, SNHG4	The low-risk group showed better OS, an independent HCC prognostic indicator. A nomogram combining features and TNM stages was constructed. TP53 mutations were more frequent in high-risk individuals. CD4+ T cells, NK cells, and macrophages differed significantly between groups. High-risk individuals had increased immune checkpoint molecule expression. The low-risk group responded better to sorafenib and cisplatin.	This study provides promising insights into DDR-associated lncrnas in HCC and provides a personalized predictive tool for prognosis and treatment response.	(125)
AC022007.1, AC023090.1, AC099850.4, ACSL6-AS1, CYTOR, LHFPL3-AS2, AL365361.1, LINC02362, and MSC-AS1	The proportion of Tregs and M0 and M2 macrophages in high-risk group was higher.	These lncRNAs may influence mitochondrial function, ferroptosis, and immune cell infiltration in HCC via specific pathways, offering valuable insights into HCC progression and treatment.	(126)
m6A-lncRNA	Many lncrnas are predictive risk factors for HCC prognosis, and the expression of M6A-lncrnas is significantly upregulated in tumor tissues, which can predict HCC prognosis independently of other clinical features.	m6A-lncRNA is closely linked to HCC occurrence and progression. Its prognostic model aids in predicting HCC outcomes, while m6A-lncRNA and immune cell infiltration in the TME offer new therapeutic targets, warranting further research.	(127)
Cuproptosis-related lncRNA	High CLS is associated with more aggressive tumor characteristics and treatment resistance. TP53, CSMD1, and RB1 mutations are more frequent in high CLS samples, which also show more amplifications and deletions. High CLS patients are more sensitive to 5-fluorouracil, gemcitabine, and doxorubicin, while low CLS patients respond better to immunotherapy.	In this study, we used machine learning to create CLs-based prognostic profiles and conducted comprehensive analyses in the areas of function, immunity, mutation, and clinical application.	(128)
Cuproptosis-related lncRNAs	The high-risk group had a higher TMB and a poorer prognosis, while the low-risk group had a higher activity of NK cells and less infiltration of Tregs, indicating a better prognosis.	CupRLSig serves as a prognostic tool for HCC, predicting immune invasion levels and potential tumor immunotherapy efficacy.	(129)
Cuproptosis-Related LncRNAs	The low-risk group exhibited higher infiltration of anti-tumor immune cells and greater sensitivity to ICIs, potentially mediated by the AL365361.1/hsa-miR-17-5p/NLRP3 axis. NLRP3 mutation-sensitive drugs like VNLG/124, Sunitinib, and rifampicin may benefit high-risk patients.	The crLncRNAs model has good specificity and sensitivity, and can be used to classify and treat sensitive populations and predict the prognosis of HCC patients.	(119)
Ferroptosis-related lncRNAs	A 12 ferroptosis-related lncRNA prognostic model for HCC stratifies patients into high-risk and low-risk groups, with the high-risk group showing elevated immune checkpoint molecule expressions (PD-1, CTLA-4, CD86).	This model may serve as a new indicator for the response and adverse reactions of HCC to immunotherapy.	(94)
Ferroptosis-related lncRNAs	A ferroptosis-associated lncRNA feature has been established, linked to immune checkpoint blockade targets like PD-1, CTLA-4, and TIM-3.	The three lncRNAs may act as potential therapeutic targets for HCC patients, and their profiles could aid in prognostic prediction for HCC.	(95)

### 4.2 Combination therapy

In Chapter Three, we discuss how lncRNAs can regulate the TME, influencing the infiltration and function of immune cells, thereby enhancing the efficacy of immunotherapy. Certain lncRNAs can alter the immune status of the TME by inhibiting the expression of immunosuppressive factors or promoting the release of immune-activating factors. This underscores the promising therapeutic prospects of combining lncRNA-targeted therapy with immunotherapy. For example, TUG1 can regulate the expression of PD-L1 and CD47 by acting as a “sponge” for miR-141 and miR-340. Knockdown of TUG1 can inhibit tumor growth and metastasis, increase the infiltration of CD8+ T cells and M1 macrophages in the tumor, and activate CD8+ T cells through PD-L1 while enhancing macrophage phagocytosis through CD47. Therefore, TUG1-siRNA combined with PD-L1 antibodies can effectively suppress tumor growth, providing new targets and strategies for HCCimmunotherapy (51). Additionally, various lncRNAs discussed earlier, such as MIAT (52), LINC01132 (56), MIR155HG (91), PCED1B-AS1 (64), NEAT1 (49), can serve as potential combined treatment targets to enhance the effects of HCC immunotherapy (**Figure 2D**). However, it is worth noting that, despite the promising therapeutic prospects of combination therapies, large-scale clinical trials of lncRNA-targeted drugs alone or in combination with immunotherapy for treating HCC have not yet been widely conducted.

In addition to the combination with immunotherapy, significant progress has been made in the study of lncRNA targeting drugs in combination with chemotherapy, anti-angiogenic drugs or targeted kinase inhibitors in tumors. The mechanism mainly focuses on the synergistic regulation of tumor drug resistance, angiogenesis and signaling pathways. Studies have shown that lncrnas can affect the expression of apoptosis-related genes by regulating DNA damage repair or adsorbing mirnas as ceRNA, thus reversing chemotherapy resistance (130). In addition, lncrnas can also regulate vascular endothelial growth factor (VEGF) related pathways, such as linc00173.v1, which promotes angiogenesis through the miR-511-5p/VEGFA axis (131). Other studies have shown that lncrnas recruit kinase complexes through scaffold action (e.g., WDR5/KAT2A complex regulates SOD2 gene (132)) or regulate kinase-related signaling pathways (e.g., STAT3/VEGFA axis (133)). However, current research still faces challenges, such as *in vivo* safety verification of lncRNA targeted delivery systems (such as lipid nanoparticles and viral vectors), as well as dose optimization of combination regimenes and analysis of drug resistance mechanisms. Overall, this combination strategy provides a new direction to overcome the limitations of traditional therapies by regulating the TME and molecular networks in multiple dimensions.

## 5 Future perspectives and challenges

Recent advancements in the understanding of lncRNAs have provided valuable insights into their roles within the immune microenvironment of HCC. Research has revealed that lncRNAs are not merely passive players but active regulators of immune cell behavior, influencing tumor progression and response to therapy (134). For instance, studies have highlighted specific lncRNAs that modulate T cell activity, macrophage polarization, and the overall cytokine profile within the TME (135). These findings suggest that lncRNAs can serve as critical mediators of immune evasion and resilience in HCC.

Moreover, the identification of lncRNAs as potential biomarkers for prognosis and treatment response has opened new avenues for personalized immunotherapy approaches in HCC (136). By integrating lncRNA profiling into clinical practice, oncologists may better predict patient responses to immunotherapeutic strategies and tailor treatments accordingly (94, 137). This perspective shift emphasizes the potential for lncRNAs to enhance the efficacy of existing therapies and improve patient outcomes.

Despite the promising insights gained from current research, several challenges remain in translating lncRNA findings into clinical practice. One of the primary obstacles is the complex regulatory mechanisms underlying lncRNA functions. The interactions between lncRNAs and their target genes, as well as the pathways they influence, can be intricate and context-dependent, complicating efforts to target them effectively (138, 139).

Additionally, the identification of specific lncRNA targets poses another challenge. Given the pleiotropic effects of many lncRNAs, achieving specificity in targeting their functions can be difficult (140). This challenge underscores the need for advanced methodologies to elucidate lncRNA interactions and their precise roles within the HCC immune microenvironment.

Drug delivery systems also represent a significant hurdle in lncRNA-based therapies. Ensuring efficient and targeted delivery of lncRNA modulators to tumor tissues while minimizing off-target effects is critical for maximizing therapeutic efficacy (141). Advances in nanotechnology and RNA delivery systems may provide solutions to these challenges, enabling more effective clinical applications of lncRNA therapeutics (142–144).

Although most current studies focus on lncRNAs as biomarkers, some lncRNAs play regulatory roles in the tumor immune microenvironment that render them potential direct therapeutic targets. For example, lncRNA NEAT1 promotes immune evasion by modulating PD-L1 expression, thereby affecting the sensitivity of tumor cells to immunotherapy; inhibition of NEAT1 may enhance immunotherapeutic efficacy (145–147). Similarly, lncRNA MALAT1 influences T cell infiltration and augments tumor immune suppression by modulating the STAT3 signaling pathway (148, 149). In addition, antisense oligonucleotides (ASOs) targeting MALAT1 have demonstrated promising therapeutic potential (150, 151). Moreover, lncRNA UCA1 regulates PD-L1 expression via miR-148a, and its inhibition could improve the efficacy of ICIs (152). Therefore, the design of lncRNA-targeted intervention strategies—such as RNAi, ASOs, or CRISPR-Cas13-mediated lncRNA degradation—could represent a novel immunotherapeutic approach (153–155).

Despite the potential of lncRNAs as therapeutic targets, several challenges remain. Many lncRNAs exhibit tissue- or cell type-specific expression, and strategies to precisely target specific cells without affecting normal tissues need further exploration. Furthermore, the complex structure of lncRNAs, with some harboring highly stable secondary structures, makes them difficult to degrade or inhibit effectively. Although *in vitro* studies often demonstrate promising intervention outcomes, *in vivo* delivery of RNA-based therapeutics faces challenges including degradation, immunogenicity, and hepatic clearance.

Various RNA delivery strategies have been developed; however, systems optimized specifically for lncRNAs still require refinement. LNPs, which have been approved by the FDA for mRNA vaccine delivery, can be used for lncRNA modulators but still need improvements in tissue specificity (156, 157). Viral vectors, such as AAVs, can facilitate the stable expression of shRNAs to knock down pathogenic lncRNAs, although they may trigger immune responses (158). Chemical modifications like 2’-O-methylation and locked nucleic acid modifications can enhance the stability of lncRNA interference molecules (159, 160).

Meanwhile, the off-target effects of lncRNA modulators should not be overlooked. Many lncRNAs possess highly homologous sequences, so interference with a specific lncRNA may inadvertently affect other non-target RNAs, leading to unexpected biological effects (161, 162). Additionally, lncRNAs may interact with miRNAs through ceRNA mechanisms, whereby targeting a particular lncRNA could trigger cascade effects in miRNA-associated pathways (160). To evaluate such off-target phenomena, techniques such as single-cell sequencing, RNA pull-down assays, and RBP interaction studies can be employed to assess the specificity of lncRNA modulators (163, 164).

Previous studies, as summarized in this review, have shown that the potential of lncRNAs in HCC immunotherapy has been validated in several preclinical studies, demonstrating their significant potential in regulating the immune microenvironment, promoting immune evasion, and serving as therapeutic targets. However, these findings are still far from mature and lack sufficient clinical validation. Currently, most related studies remain in the basic and preclinical research stages, with a lack of clinical trials targeting lncRNA-based therapies for HCC. For example, ASOs targeting MALAT1 have shown preliminary therapeutic effects in mouse models, but their clinical efficacy still needs further confirmation (151, 165). Similarly, although interventions targeting PVT1 and HOTAIR have shown promising results in preclinical studies, there is still a lack of relevant clinical data to assess their therapeutic potential (166). Furthermore, it is important to acknowledge the many challenges in implementing lncRNA-targeted therapeutic strategies, such as those mentioned earlier regarding the specificity of drug delivery to lncRNAs and the avoidance of systemic toxicity, as well as off-target effects that may cause adverse reactions. Therefore, before advancing lncRNA-targeted therapies into clinical practice, these technical barriers must be addressed, and their safety and efficacy should be validated through more extensive clinical trials. As a result, research on lncRNAs as immune therapy targets remains in its early stages. Despite their potential demonstrated in preclinical experiments, there are still many technical and clinical challenges to overcome before applying them in clinical settings. Future research should place greater emphasis on critically evaluating the feasibility and limitations of these therapeutic strategies and strengthening clinical validation efforts.

Looking ahead, future research directions may focus on the development of lncRNA-based therapies that can be integrated into existing treatment regimens. Exploring combination therapies that leverage the immune-modulating effects of lncRNAs alongside ICIs or targeted therapies could yield synergistic benefits. Furthermore, the continued investigation of lncRNA dynamics in response to various treatment modalities will be crucial for optimizing therapeutic strategies.

In summary, while the field of lncRNAs in HCC presents exciting opportunities for enhancing our understanding of tumor-immune interactions, significant challenges remain. Continued research efforts will be essential to unravel the complexities of lncRNA functions and translate these findings into effective clinical interventions.
